# Altering phosphoinositides in high‐fat diet‐associated prostate tumor xenograft growth

**DOI:** 10.1002/mco2.89

**Published:** 2021-10-28

**Authors:** Mingguo Huang, Atsushi Koizumi, Shintaro Narita, Hiroki Nakanishi, Hiromi Sato, Soki Kashima, Taketoshi Nara, Sohei Kanda, Kazuyuki Numakura, Mitsuru Saito, Shigeru Satoh, Hiroshi Nanjo, Takehiko Sasaki, Tomonori Habuchi

**Affiliations:** ^1^ Department of Urology Akita University Graduate School of Medicine Akita Japan; ^2^ Research Center for Biosignal Akita University Graduate School of Medicine Akita Japan; ^3^ Department of Clinical Pathology Akita University Graduate School of Medicine Akita Japan; ^4^ Department of Biochemical Pathophysiology/Lipid Biology Medical Research Institute Tokyo Medical and Dental University Bunkyo‐ku Tokyo Japan

**Keywords:** AKT, FASN, high‐fat diet, phosphoinositide, prostate cancer

## Abstract

The metabolic reprogramming of phospholipids may affect intracellular signal transduction pathways. A high‐fat diet (HFD) is attributed to prostate cancer (PCa) progression, but the expression pattern and role of phospholipids in HFD‐mediated PCa progression remains unclear. In this study, HFD enhanced LNCaP xenograft tumor growth by upregulating the phosphatidylinositol (PI) 3‐kinase (PI3K)/AKT signaling pathway. A lipidomic analysis using xenograft tumors showed that phosphoinositides, especially PI (3,4,5)‐trisphosphate (PIP_3_), including several species containing C38:4, C38:3, and C40:4 fatty acids, increased in the HFD group compared to control. Fatty acid synthase (FASN) was significantly upregulated in xenograft tumors under HFD in both gene and protein levels. PCa cell growth was significantly inhibited through the decreased AKT signaling pathway by treatment with cerulenin, a chemical FASN inhibitor, which also downregulated PIP, PIP_2_, and PIP_3_ but not PI. Thus, dietary fat influences PCa progression and alters phosphoinositides, especially PIP_3_, a critical player in the PI3K/AKT pathway. These results may offer appropriate targets, such as FASN, for dietary intervention and/or chemoprevention to reduce PCa incidence and progression.

## Graphical abstract

HFD‐induced alteration of PIPs, especially PIP_3_, may activate proliferative signaling transduction, such as the PI3K/AKT pathway through the upregulation of FASN, and influence PCa progression. Therefore, PI3K/AKT signaling and FASN could be the important targets for dietary intervention and/or chemoprevention in PCa incidence and progression.

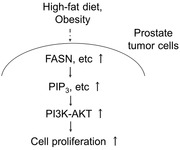

## 1 INTRODUCTION

Prostate cancer (PCa) is one of the most common cancers in men. Epidemiologic studies have indicated that dietary fat is a vital environmental risk factor in PCa incidence and progression,^1^, ^2^, ^3^ although the underlying molecular mechanisms are still not fully understood. Lipidomic analyses have reported that high‐fat diet (HFD) consumption induces substantial changes in multiple lipid classes in various organs, including prostate phospholipids.^4^ Dietary fat can influence PCa progression by modifying gene expression and bioactive mediators that activate intracellular signaling pathways, such as AKT and mitogen‐activated protein kinase signaling pathways, to stimulate tumor cell growth and survival.^5^, ^6^, ^7^, ^8^ Additionally, upregulated de novo lipogenesis is known to protect PCa cells from radiation and chemotherapeutics.^9^, ^10^, ^11^ Fatty acid synthase (FASN), a key enzyme that plays a critical role in the long‐chain saturated fatty acid synthesis, is frequently upregulated during PCa onset and metastatic progression and is regarded as an unfavorable prognosis factor in PCa patients.^12^ Thus, exogenously consumed or endogenously synthesized lipids play key roles in the developing PCa stages.

Phosphatidylinositol (PI) is a unique phospholipid that can be phosphorylated by lipid kinases. PI mono‐, bis‐, and trisphosphates (PIP, PIP_2_, and PIP_3_, respectively) are collectively referred to as phosphoinositides. These membrane lipids interact with effector proteins by their phosphorylated inositol head groups, thereby regulating many critical and common intracellular signal transduction pathways typically upregulated during cancer incidence and progression.^13^ Several studies have reported aberrant alterations in lipid molecules in cancerous cells and tissue. However, little is known about phosphoinositide profiles in many cancers.^14^ This is due to the technical limitations of the conventional lipidomic approach, as phosphoinositides are out of its coverage range. Therefore, phosphoinositide analysis is more complicated than other phospholipid classes because these molecules have multiple phosphate moieties and are in very low abundance in cell lines and animal tissue lipid extracts. A specialized method is required to address the possible dietary fat effects on phosphoinositide dynamics.

This study examined the phosphoinositides levels in human LNCaP PCa xenografts in mice using a targeted lipidomic method. HFD increased PIP_3_ levels and enhanced tumor growth. Mechanistically, HFD‐mediated FASN upregulation plays an essential role in phosphoinositide metabolism and cell proliferation.

## 2 RESULTS

### 2.1 HFD enhanced tumor growth and AKT activation in LNCaP mice xenografts

**FIGURE 1:**
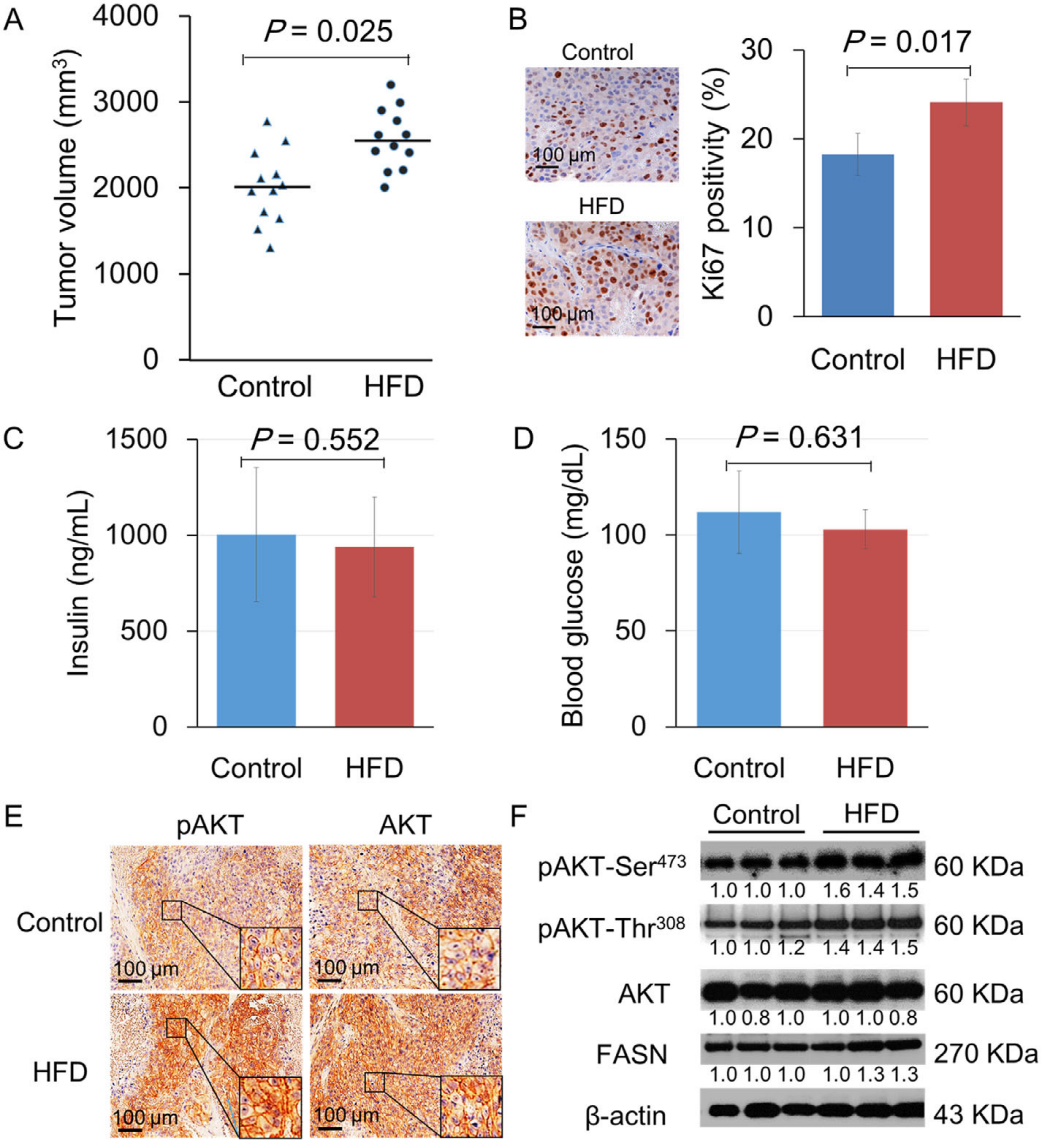
High‐fat diet (HFD) enhanced tumor growth in LNCaP xenograft mice with the upregulated PI3K/AKT pathway. LNCaP xenograft mice were subcutaneously injected with tumor cells and fed with HFD or control diet. On week 14, mice were sacrificed, and xenograft tumors were excised to performed lipid analysis. (A) The tumor volume level was enhanced in the HFD group compared to control. (B) Xenograft tumor sections were subjected to anti‐Ki‐67 staining. Ki‐67 positivity was significantly higher in the HFD group than the control group. Bar, 100 μm. (C) The serum insulin level, as measured by the specific ELISA kit, was no statistical difference in the diet groups. (D) Mouse blood glucose levels were quantified using LAB Gluco. There was no significant difference in blood glucose levels between the diet groups. (E) Xenograft tumor sections from mice in the two diet groups underwent immunohistological staining with anti‐human pAKT (Ser^473^) and pan‐AKT antibodies. The expression was semiquantitatively calculated. pAKT staining intensities were significantly higher in the HFD group than the control group. Bar, 100 μm. (F) pAKT (Ser^473^ and Thr^308^), AKT, and FASN expression in lysates of individual LNCaP xenograft tumors (three samples each group) was detected by Western blot using specific antibodies. β‐actin was used as an intrinsic control

At 14 weeks of the prostate tumor xenograft study, tumor growth was significantly increased in mice fed with HFD than the control diet (2569.9 ± 354.7 vs. 2010.3 ± 428.7 mm^3^; *p* = 0.025; Figure 1A). Body weight (22.3 ± 1.9 vs. 19.8 ± 2.1 g; *p* = 0.147) and caloric intake (10.9 vs. 9.3 kcal/day; *p* = 0.083) were also higher in the HFD group than the control group. Ki‐67 expression levels, a cell proliferation marker, in xenograft tumor tissue were examined by immunohistochemistry. The proportion of Ki‐67‐positive cells was significantly higher in the HFD group than the control group (24.1 ± 2.6% vs. 18.3 ± 2.4%; *p* = 0.017; Figure 1B), showing enhanced cancer cell proliferation in mice fed with HFD. In addition, we compared the levels of serum insulin and blood glucose between the diet groups. The mean serum insulin levels were 111.9 ± 21.5 and 102.9 ± 10.3 mg/dl (*p* = 0.562), and the mean blood glucose levels were 1003.4 ± 350.3 and 938.7 ± 261.4 ng/ml (*p* = 0.631) in the two diet groups, respectively (Figure 1C and 1D).

To gain insights into the tumor growth promotion mechanism by HFD, this study focused on AKT (also known as protein kinase B). This serine/threonine kinase plays a critical role in regulating diverse cell growth and survival processes. Previous studies have reported that AKT is activated in PCa xenografts upon HFD feeding. Consistent with previous studies, increased AKT phosphorylation at Ser^473^ and plasma membrane recruitment (hallmarks of AKT activation) were observed in xenograft tissue receiving HFD (Figure 1E). Immunohistochemistry using a pan‐AKT antibody also demonstrated that AKT showed plasma membrane localization. Similarly, Western blot analysis has shown that HFD consumption also enhanced pAKT (Ser^473^ and Thr^308^) and FASN expression in xenograft tumors (Figure 1F).

### 2.2 HFD affected phosphoinositide metabolism in LNCaP xenograft tissue

**FIGURE 2:**
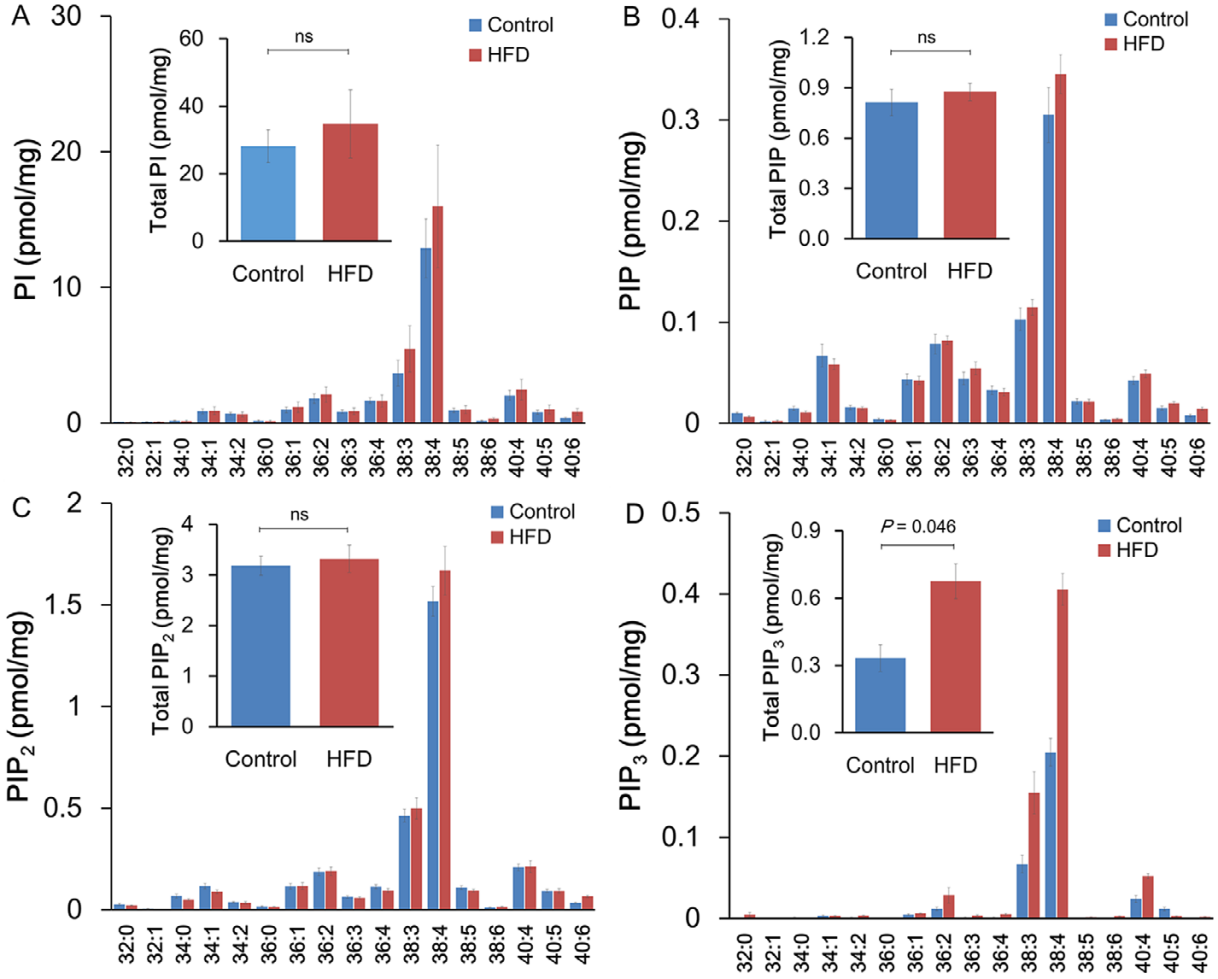
High‐fat diet (HFD) affected phosphoinositide metabolism in LNCaP xenograft tissue. (A–D) Total lipids from mouse xenograft tumor tissue (10 mg; seven mice per group) were extracted. Total PI (A), PIP (B), PIP_2_ (C), and PIP_3_ (D) were quantified by liquid chromatography‐mass spectrometry (LC‐MS)/MS analysis. Abbreviation: ns, not significant.

To examine how HFD induces AKT hyperactivation in LNCaP xenograft tissue, tumor phosphoinositides were measured using reverse‐phase liquid chromatography (LC)‐mass spectrometry (MS) (Figure 2). The 17 major molecular species with different fatty acyl chain combinations were analyzed for each phosphoinositide class. There was no difference in the PI, PIP, or PIP_2_ levels between HFD and control groups. The total level comparisons (sum of all molecular species) of PI (28.2 ± 4.48 vs. 34.8 ± 9.35 pmol/mg; Figure 2A), PIP (0.81 ± 0.07 vs. 0.88 ± 0.05 pmol/mg; Figure 2B), and PIP_2_ (3.18 ± 0.17 vs. 3.31 ± 0.25 pmol/mg; Figure 2C) also did not show any differences between the diet groups. In contrast, the relatively abundant PIP_3_ was increased when mice were fed with HFD, namely C38:4, C38:3, and C40:4 PIP_3_. This resulted in a twofold increase in the total PIP_3_ (0.33 ± 0.06 vs. 0.69 ± 0.14 pmol/mg; *p* < 0.05; Figure 2D). These data demonstrated for the first time that dietary fat intake alters the phosphoinositide phosphorylation status.

### 2.3 FASN was upregulated in LNCaP xenograft tumors under HFD feeding and regulated PCa cell growth and pAKT expression

**FIGURE 3:**
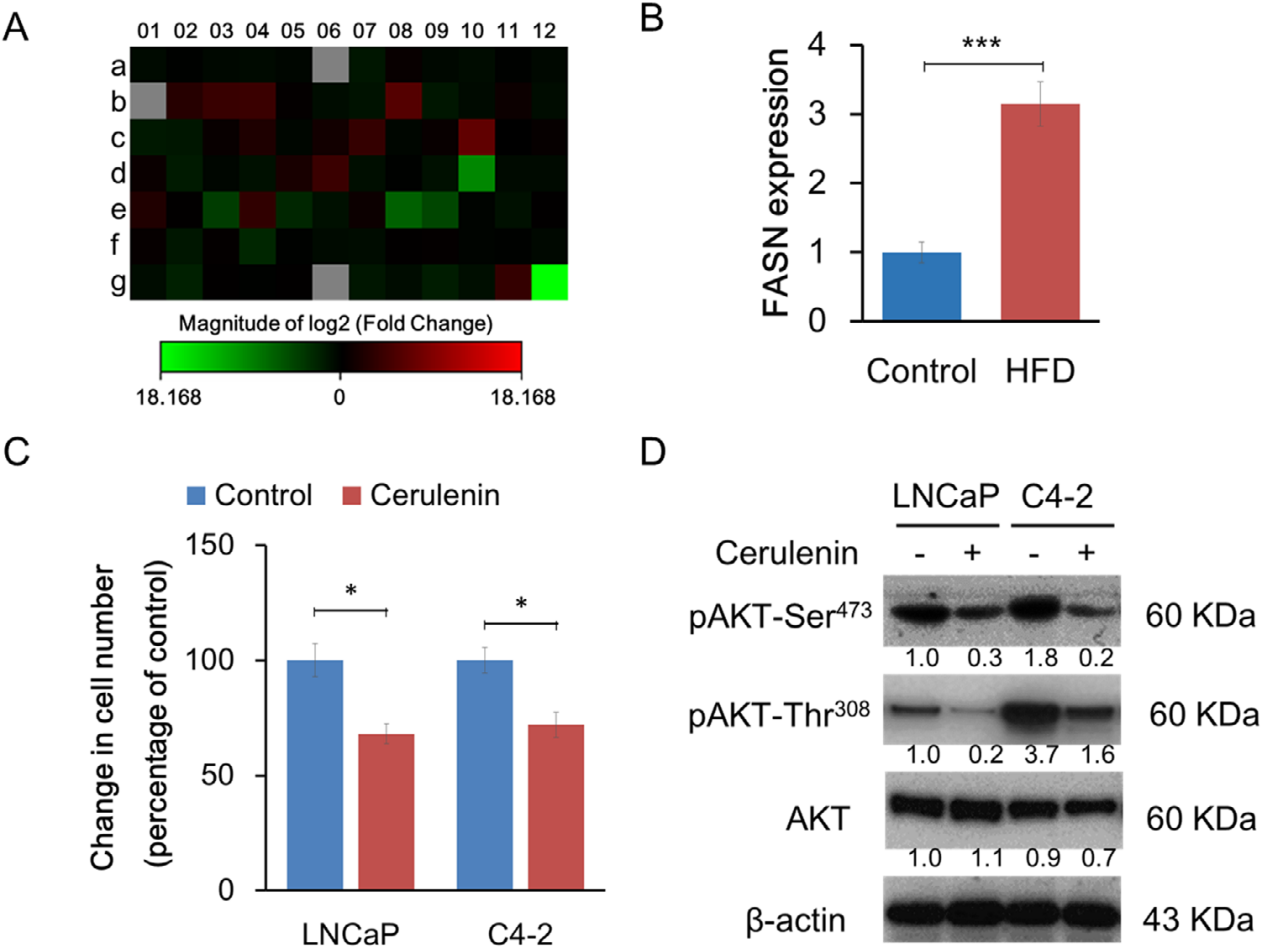
Fatty acid synthase (FASN) was upregulated in LNCaP xenograft tumors under HFD feeding and regulated cell growth and pAKT expression in PCa cells. The expression of 84 key genes involved in regulating fatty acid metabolism was comprehensively analyzed by a Human Fatty Acid Metabolism PCR Array kit using xenograft tumors. (A) The cluster heat map shows the relative gene expression between the diet groups. The assay was performed in duplicate within each group. Wells a1 to g12 each contain real‐time PCR array for the fatty acid metabolism‐related gene. Increased levels are red, and decreased levels are green. (B) FASN was significantly upregulated in xenograft tumors under HFD feeding. (C and D) Role of FASN in PCa cell viability and AKT expression. LNCaP and C4‐2 cells were cultured with or without 10 μM cerulenin for 24 h. (C) Cell proliferation was assessed using a nonradioactive MTT‐based Cell Proliferation Assay kit, and cell viability in cells treated with cerulenin was compared to untreated cells. *p < 0.05. (D) pAKT (Ser^473^ and Thr^308^) and AKT expression in lysates (10 μg) were detected by Western blot using specific antibodies. β‐actin was used as an intrinsic control. ***p < 0.001

A human fatty acid polymerase chain reaction (PCR) array was conducted to define the lipogenic gene expression levels in xenograft tumors (Figure 3A). When differently expressed genes under the two diet conditions were filtered by more than twofold changes, 17 genes were upregulated, and 20 genes were downregulated in HFD xenograft tumors compared to control. The upregulated genes included acetyl‐CoA‐related enzymes, CoA reductases, fatty acid‐binding molecules, and FASN (Table S1; Figure 3B). FASN is the key enzyme that catalyzes the terminal steps to synthesize long‐chain fatty acids and is directly regulated by androgen receptor (AR), and its expression levels are closely associated with PCa cell proliferation.^12^, ^15^ Enhanced FASN protein expression in the HFD xenograft tumor group was also confirmed by Western blot, as shown in Figure 1D

Consistent with previous studies, cerulenin, an FASN‐specific inhibitor, suppressed LNCaP cell proliferation through the decreased AKT pathway *in vitro* (Figure 3C,D). This inhibitory effect was also observed in C4‐2 cells, an LNCaP subline that lost androgen responsiveness due to lower AR protein and mRNA expression levels. This suggested that FASN positively regulates cell proliferation through an androgen‐independent and cell autonomous mechanism that affects the AKT pathway.

### 2.4 FASN differently regulated phosphoinositides in PCa cells

**FIGURE 4:**
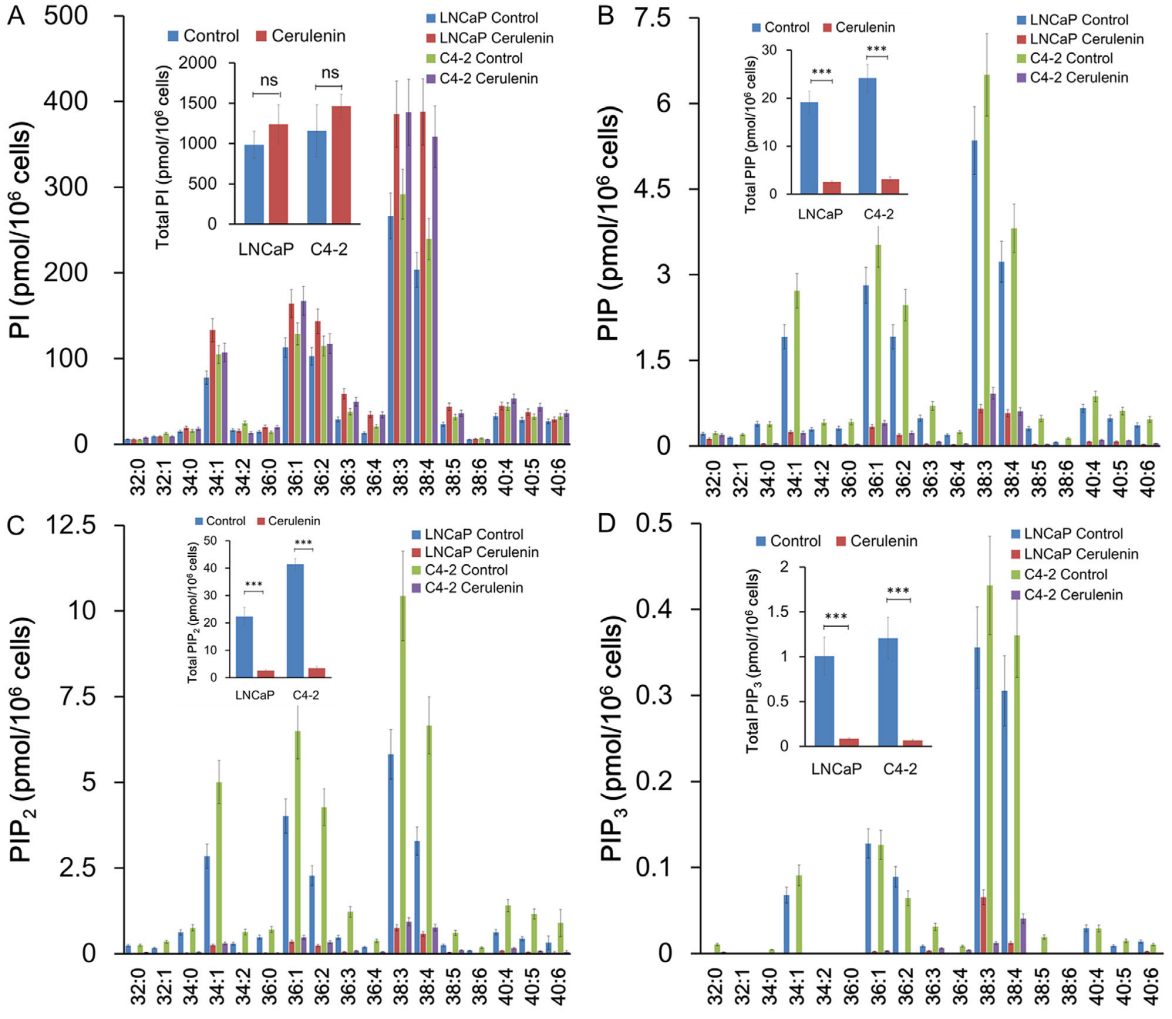
FASN regulated phosphoinositides in PCa cells. (A–D) LNCaP and C4‐2 cells (1 × 10^6^) were seeded in a 60‐mm dish and treated with 10 μM cerulenin for 24 h. Total lipids from cultured cells were extracted, and PI and PIPs were quantified. The expression levels of PIP (B), PIP_2_ (C), and PIP_3_ (D), but not PI (A), were decreased in PCa cells treated with cerulenin compared to untreated cells. ***p < 0.001

**FIGURE 5:**
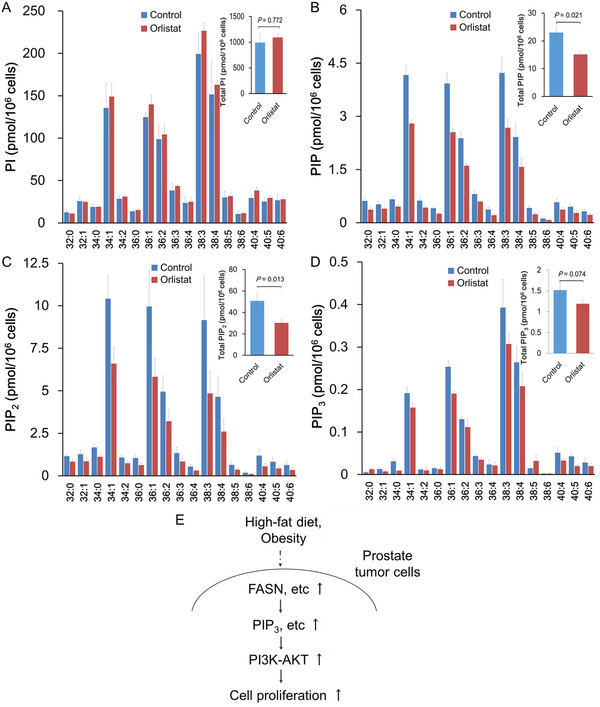
Orlistat decreased PIPs levels but not PI in PCa C4‐2 cells. (A–D) C4‐2 cells (1 × 10^6^) were seeded in a 60 mm dish and treated with 10 μM orlistat for 24 h. Total lipids from cultured cells were extracted, and PI and PIPs were quantified. The expression levels of PIP (B), PIP_2_ (C), and PIP_3_ (D), but not PI (A), were decreased in PCa cells treated with orlistat compared to untreated cells. (E) Schematic of the putative effect of HFD and/or obesity on the PCa progression. HFD‐induced alteration of PIPs, especially PIP_3,_ may activate proliferative signaling transduction, such as the PI3K/AKT pathway through the upregulation of FASN, and influence PCa progression. Thus, PI3K/AKT signaling and FASN could be the important targets for dietary intervention and/or chemoprevention in PCa incidence and progression

The effects of cerulenin on cellular phosphoinositide levels were examined. Cerulenin increased the levels of several PI species (Figure 4). In contrast, cerulenin caused a significant drop in PIP, PIP_2_, and PIP_3_ levels in both LNCaP and C4‐2 cells (Figure 4A–D). The effect was greatest on PIP_3_, then PIP_2_, and finally PIP. These data demonstrated that FASN is required for normal PI phosphorylation at D4‐, D5‐, and D3‐hydroxyl of the inositol ring, which sequentially produces PIP, PIP_2_, and PIP_3_. However, FASN is dispensable for de novo PI synthesis. Similar to the effects of cerulenin, the PIP, PIP_2_, and PIP_3_, but not PI, levels were decreased in PCa C4‐2 cells with orlistat treatment, a mild inhibitor of FASN (Figure 5A‐D).

## 3 DISCUSSION

AKT has a pleckstrin homology domain that recruits it to the cell membrane and is enriched in PIP_3_, the lipid product of PI 3‐kinase (PI3K). This feature is critical for AKT activation because PIP_3_ recruits phosphoinositide‐dependent kinase 1 (PDK1) and facilitates the PDK1‐dependent phosphorylation of AKT at the membrane compartment.^13^ Many studies have shown that HFD‐mediated secretion of cytokines and growth factors can upregulate the PI3K/AKT pathway in tumor cells.^8^, ^16^ Conversely, diet and/or calorie restriction may reduce the circulating levels of growth factors, anabolic hormones, and inflammatory cytokines associated with decreased prostate malignancies through downregulation of intrinsic signal pathways.^17^, ^18^ This study showed that HFD‐mediated upregulation of AKT signaling in PCa xenograft tumors might be a critical consequence of metabolism‐modified phosphoinositides, especially PIP_3_. As shown in the Figure 5E, HFD‐mediated lipid metabolism may provide several tumorigenic metabolites that affect signaling transduction networks for aggressive PCa progression.

Through human fatty acid metabolism PCR array analysis of xenograft tumor tissue, this study identified several genes involved in phospholipid metabolism, such as *FASN*, the main target of PCa cell metabolism. FASN is a key multienzyme for the terminal catalytic step in de novo fatty acid biogenesis.^19^ FASN is primarily expressed in normal cells at a low level and highly expressed in cancer cells and preneoplastic lesions, including PCa, with high lipid metabolism.^19^ As evidence of the oncogenic role of FASN, the transient overexpression of ectopic FASN increased the proliferation, invasion, and metastasis of immortalized human prostate epithelial cells.^12^ In this study, HFD consumption significantly upregulated FASN expression in gene and protein levels and subsequently stimulated the AKT signaling pathway in LNCaP xenograft tumors. Interestingly, the relative abundance of saturated fatty acid metabolism may be related to the human PCa state.^20^ Although more in vivo evidence is needed to prove how FASN is linked to the AKT pathway, at least the chemical inhibition of FASN significantly downregulated PIP_3_ and subsequently suppressed pAKT expression.

This study investigated the phosphoinositide dynamics in PCa cells in vitro and in the xenograft tumor model and found that HFD increases the PI3K activity lipid product PIP_3_. There were concomitant increases in the levels of oncogenic phosphoinositides and FASN expression in PCa xenograft tumors upon HFD feeding. Pharmacological PI3K activity inhibition was previously demonstrated to suppress FASN expression in PCa cells.^21^ Considering these studies, HFD may upregulate FASN expression through PI3K activation and PIP_3_ accumulation. Conversely, phosphoinositide measurements in this study revealed that FASN inhibition reduced cellular PIP_3_ in vitro. Thus, these data suggested that excess lipid intake drives a positive feedback loop to enhance and sustain the PI3K/PIP_3_ signaling pathway activity in PCa cells, stimulating cell proliferation and accelerating tumor growth. Thus, the regulatory axis provides new insights into a dietary intervention to reduce PCa incidence and progression. However, it is unclear at what frequency and to what extent this feedback loop operates in human PCa. A comprehensive analysis of PIP_3_ gene profiling should answer the question and provide a rational basis for selecting patients who will benefit from PI3K inhibitor therapy.

## 4 MATERIALS AND METHODS

### 4.1 Cell culture and reagents

The human PCa cell line LNCaP was purchased from the American Type Culture Collection (Manassas, VA, USA), and C4‐2 cells were kindly provided by Dr. Leland W.K. Chung of Emery University.^22^ Cells were maintained in RPMI 1640 or Dulbecco's modified Eagle's medium (DMEM) (Invitrogen, Carlsbad, CA, USA) containing 10% fetal bovine serum (FBS) and 1% penicillin‐streptomycin. FASN inhibitors (cerulenin and orlistat) were purchased from Sigma (St. Louis, MO, USA) and used at 10 μM concentration.

### 4.2 Animal study

The institutional review board of the Akita University Graduate School of Medicine approved all animal experiments. Six‐week‐old athymic BALB/c‐nu/nu male mice were obtained from Japan SLC (Shizuoka, Japan). LNCaP cells (1 × 10^6^) were injected with 0.25 ml ice‐cold BD Matrigel (BD Biosciences, Bedford, MA, USA) and 0.25 ml RPMI 1640. Four weeks after injection, mice with a palpable tumor were randomly assigned to two different diet groups (12 mice per group): high‐fat group (HFD) and control diet group (control). The energy composition of HFD was 59.9% fats, 21.4% carbohydrates, and 18.6% proteins, whereas the control group included 9.5% fats, 67.7% carbohydrates, and 22.8% proteins (Purina Mills Test Diets, Richmond, IN, USA). Body weight and tumor volume were measured weekly, and tumor volume was calculated as reported previously.^5^ On week 14, mice were sacrificed, xenograft tumors were excised, and lipid analysis was performed. Mouse serum insulin levels were measured by the specific Enzyme‐Linked Immunosorbent Assay (ELISA) kit (Morinaga Institute of Biological Science, Tokyo, Japan). Mouse blood glucose levels were quantified by LAB Gluco (ForaCare Japan Co., Ltd., Tokyo, Japan).

### 4.3 Lipid preparation and quantification

Total lipids from mouse xenograft tumor tissue (10 mg; seven mice per group) and cultured cells (1  ×  10^6^) were extracted using the Bligh‐Dyer method.^23^ The total lipid extract was dried under a gentle stream of nitrogen gas and redissolved in methanol. Total phosphoinositides and molecular species were quantified by LC‐tandem MS (MS/MS) analysis using a 4000 Q‐TRAP quadrupole linear ion‐trap hybrid MS (Thermo Fisher Scientific, Tokyo, Japan) with an Acquity Ultra Performance LC (Waters) according to the method reported previously.^20^

### 4.4 Human fatty acid metabolism analysis

Total RNA was extracted from LNCaP xenograft tumors using TRIzol reagent (Invitrogen). The expression of 84 key genes involved in the regulation and enzymatic pathways of fatty acid metabolism was comprehensively analyzed by the Human Fatty Acid Metabolism PCR Array kit (Qiagen, Valencia, CA, USA). The first‐strand cDNA was briefly synthesized using 1.0 μg xenograft tumor RNA from the two diet groups (two samples per group) by an RT2 First‐Strand kit (SABiosciences, Frederick, MD, USA) according to the manufacturer's instructions. Fatty acid metabolism‐focused gene expression analyses were performed using RT2 Profile PCR Arrays (Qiagen) according to the manufacturer's instructions, and the relative gene expression between the diet groups was compared.

### 4.5 Cell proliferation assay

Cells (1 × 10^4^) were seeded in a 96‐well plate and cultured in DMEM containing 2% FBS without antibiotics and treated with 10 μM cerulenin for 24 h. Cell proliferation was assessed using a methyl thiazolyltetrazolium (MTT)‐based Cell Proliferation Assay kit (Roche, Basel, Switzerland) according to the manufacturer's instructions. Absorbance was measured at 570 nm using an ELISA reader (Bio‐Rad, Tokyo, Japan). All experiments were conducted in triplicate.

### 4.6 Western blotting

Proteins were extracted from xenograft tumors and cultured cells using Complete Lysis‐M buffer (Roche). Equal amounts (10 μg) of proteins were separated by sodium dodecyl sulfate‐polyacrylamide gel electrophoresis and transferred to polyvinylidene difluoride membrane (ATTO Instruments, Tokyo, Japan). The membrane was blocked for 1 h at room temperature using 1% nonfat dry milk in phosphate‐buffered saline containing 0.1% Tween 20 and incubated for 1 h at room temperature in anti‐FASN (BD Biosciences), anti‐AKT (Cell Signaling Technology, Beverly, MA, USA), anti‐pAKT (Ser^473^ and Thr^308^; Cell Signaling Technology), and anti‐β‐actin (Cell Signaling Technology) antibody diluted in blocking buffer (1:1000). Membranes were washed and incubated with goat anti‐mouse IgG or goat anti‐rabbit IgG with horseradish peroxidase (HRP) (1:3000; Santa Cruz Biotechnology, Dallas, TX, USA). Proteins were visualized using an enhanced chemiluminescence system (Amersham Biosciences, Piscataway, NJ, USA), and the bands were quantified using CS Analyzer (2.0) software (ATTO Instruments).

### 4.7 Immunohistochemistry

Sections of formalin‐fixed, paraffin‐embedded xenografts were stained with anti‐AKT (1:100) and anti‐pAKT (Ser^473^; 1:100). After overnight incubation, tissue sections were incubated with HRP‐labeled anti‐mouse or anti‐rabbit antibody (1:5000). The staining intensity in xenograft cancer cells was scored on a semiquantitative scale, and evaluation and scoring were performed by the investigators (MH and HN). Xenograft tumor sections were treated with anti‐Ki‐67 (1:800; Cell Signaling Technology), and the Ki‐67 expression level was evaluated by counting Ki‐67‐positive cells from 400–500 tumor cells in the area containing the highest density of Ki‐67‐labeled tumor cells on the slides.

### 4.8 Statistical analysis

Statistical analyses were performed using Microsoft Excel and SPSS version 12. The statistical significance of the differences between the groups in each experiment was evaluated using unpaired Student's *t‐*test or analysis of variance. Differences were considered significant if *p* < 0.05.

## CONFLICT OF INTEREST

The authors declare that they have no conflict of interest.

## ETHICS APPROVAL

The Institutional Review Board of the Akita University School of Medicine approved all experiments. Animal studies were approved by the Committee for Ethics in Animal Experimentation of Akita University School of Medicine and were performed according to the Guideline for Animal Experiments.

## AUTHOR CONTRIBUTIONS

M.H., S.N., T.S. and T.H. conceived the project and designed experiments. M.H., H.N., A.K. and H.N. performed the experiments. M.H., S.N., H.N., H.S., S.K., A.K., T.N., S.K., K.N., M.S., S.S. H.N., T.S. and T.H. analyzed the data. M.H. and T.S. wrote the manuscript.

## Supporting information

Table S1

## Data Availability

The data used during the current study are available from the corresponding author on reasonable request.
