# Supplementary material for: Altering phosphoinositides in high‐fat diet‐associated prostate tumor xenograft growth
Source: MedComm (2020). 2021 Oct 28;2(4):756–64. doi: 10.1002/mco2.89 (PMC8706770; doi:10.1002/mco2.89)
Supplement: Supplementary file 1 — Table S1 [file MCO2-2-756-s001.doc]

**Supplementary Table S1. Fold changes of genes in the xenograft under HFD compared with that of control diet.**

| **Gene Symbol** | **Description** | **Mean Fold Change** |
| --- | --- | --- |
| ACAA1 | Acetyl-CoA acyltransferase 1 | -1.70 |
| ACAA2 | Acetyl-CoA acyltransferase 2 | -1.15 |
| ACAD10 | Acyl-CoA dehydrogenase family, member 10 | -1.55 |
| ACAD11 | Acyl-CoA dehydrogenase family, member 11 | -1.66 |
| ACAD9 | Acyl-CoA dehydrogenase family, member 9 | -1.35 |
| ACADL | Acyl-CoA dehydrogenase, long chain | 7.53 |
| ACADM | Acyl-CoA dehydrogenase, C-4 to C-12 straight chain | -2.88 |
| ACADS | Acyl-CoA dehydrogenase, C-2 to C-3 short chain | 1.47 |
| ACADSB | Acyl-CoA dehydrogenase, short/branched chain | -1.43 |
| ACADVL | Acyl-CoA dehydrogenase, very long chain | -1.85 |
| ACAT1 | Acetyl-CoA acetyltransferase 1 | -1.09 |
| ACAT2 | Acetyl-CoA acetyltransferase 2 | -1.42 |
| ACOT1 | Acyl-CoA thioesterase 1 | 7.53 |
| ACOT12 | Acyl-CoA thioesterase 12 | 6.55 |
| ACOT6 | Acyl-CoA thioesterase 6 | 16.36 |
| ACOT7 | Acyl-CoA thioesterase 7 | 18.03 |
| ACOT8 | Acyl-CoA thioesterase 8 | 1.23 |
| ACOT9 | Acyl-CoA thioesterase 9 | -1.74 |
| ACOX1 | Acyl-CoA oxidase 1, palmitoyl | -2.39 |
| ACOX2 | Acyl-CoA oxidase 2, branched chain | 60.63 |
| ACOX3 | Acyl-CoA oxidase 3, pristanoyl | -2.88 |
| ACSBG1 | Acyl-CoA synthetase bubblegum family member 1 | -1.61 |
| ACSBG2 | Acyl-CoA synthetase bubblegum family member 2 | 1.86 |
| ACSL1 | Acyl-CoA synthetase long-chain family member 1 | -1.79 |
| ACSL3 | Acyl-CoA synthetase long-chain family member 3 | -3.22 |
| ACSL4 | Acyl-CoA synthetase long-chain family member 4 | -2.94 |
| ACSL5 | Acyl-CoA synthetase long-chain family member 5 | 1.46 |
| ACSL6 | Acyl-CoA synthetase long-chain family member 6 | 4.57 |
| ACSM3 | Acyl-CoA synthetase medium-chain family member 3 | -1.35 |
| ACSM4 | Acyl-CoA synthetase medium-chain family member 4 | 2.35 |
| ACSM5 | Acyl-CoA synthetase medium-chain family member 5 | 13.38 |
| ALDH2 | Aldehyde dehydrogenase 2 family (mitochondrial) | -1.28 |
| BDH1 | 3-hydroxybutyrate dehydrogenase, type 1 | 1.55 |
| BDH2 | 3-hydroxybutyrate dehydrogenase, type 2 | 109.29 |
| CPT1A | Carnitine palmitoyltransferase 1A (liver) | 1.03 |
| CPT1B | Carnitine palmitoyltransferase 1B (muscle) | 1.34 |
| CPT1C | Carnitine palmitoyltransferase 1C | 1.72 |
| CPT2 | Carnitine palmitoyltransferase 2 | -3.68 |
| CRAT | Carnitine O-acetyltransferase | -1.35 |
| CROT | Carnitine O-octanoyltransferase | -2.14 |
| DECR1 | 2,4-dienoyl CoA reductase 1, mitochondrial | 3.12 |
| DECR2 | 2,4-dienoyl CoA reductase 2, peroxisomal | 17.53 |
| ECHS1 | Enoyl CoA hydratase, short chain, 1, mitochondrial | -1.97 |
| ECI2 | Enoyl-CoA delta isomerase 2 | -1.06 |
| EHHADH | Enoyl-CoA, hydratase/3-hydroxyacyl CoA dehydrogenase | -2.31 |
| FABP1 | Fatty acid binding protein 1, liver | -819.16 |
| FABP2 | Fatty acid binding protein 2, intestinal | -1.60 |
| FABP3 | Fatty acid binding protein 3, muscle and heart (mammary-derived growth inhibitor) | -1.70 |
| FABP4 | Fatty acid binding protein 4, adipocyte | 4.93 |
| FABP5 | Fatty acid binding protein 5 (psoriasis-associated) | 1.15 |
| FABP6 | Fatty acid binding protein 6, ileal | -19.40 |
| FABP7 | Fatty acid binding protein 7, brain | 10.87 |
| FASN | Fatty acid synthase | 3.15 |
| GCDH | Glutaryl-CoA dehydrogenase | -2.20 |
| GK | Glycerol kinase | 1.88 |
| GK2 | Glycerol kinase 2 | -117.62 |
| GPD1 | Glycerol-3-phosphate dehydrogenase 1 (soluble) | -32.63 |
| GPD2 | Glycerol-3-phosphate dehydrogenase 2 (mitochondrial) | -1.22 |
| HADHA | Hydroxyacyl-CoA dehydrogenase/3-ketoacyl-CoA thiolase/enoyl-CoA hydratase (trifunctional protein), alpha subunit | -2.29 |
| HMGCL | 3-hydroxymethyl-3-methylglutaryl-CoA lyase | 1.13 |
| HMGCS1 | 3-hydroxy-3-methylglutaryl-CoA synthase 1 (soluble) | 1.41 |
| HMGCS2 | 3-hydroxy-3-methylglutaryl-CoA synthase 2 (mitochondrial) | -2.88 |
| LIPE | Lipase, hormone-sensitive | 1.20 |
| LPL | Lipoprotein lipase | -6.22 |
| MCEE | Methylmalonyl CoA epimerase | -1.07 |
| MUT | Methylmalonyl CoA mutase | -1.67 |
| OXCT2 | 3-oxoacid CoA transferase 2 | -1.79 |
| PECR | Peroxisomal trans-2-enoyl-CoA reductase | 1.07 |
| PPA1 | Pyrophosphatase (inorganic) 1 | 1.23 |
| PRKAA1 | Protein kinase, AMP-activated, alpha 1 catalytic subunit | -1.20 |
| PRKAA2 | Protein kinase, AMP-activated, alpha 2 catalytic subunit | -1.13 |
| PRKAB1 | Protein kinase, AMP-activated, beta 1 non-catalytic subunit | -1.46 |
| PRKAB2 | Protein kinase, AMP-activated, beta 2 non-catalytic subunit | -1.73 |
| PRKACA | Protein kinase, cAMP-dependent, catalytic, alpha | -4.88 |
| PRKACB | Protein kinase, cAMP-dependent, catalytic, beta | 1.10 |
| PRKAG1 | Protein kinase, AMP-activated, gamma 1 non-catalytic subunit | -1.14 |
| PRKAG2 | Protein kinase, AMP-activated, gamma 2 non-catalytic subunit | 1.11 |
| PRKAG3 | Protein kinase, AMP-activated, gamma 3 non-catalytic subunit | 7.53 |
| SLC27A1 | Solute carrier family 27 (fatty acid transporter), member 1 | -2.90 |
| SLC27A2 | Solute carrier family 27 (fatty acid transporter), member 2 | -1.83 |
| SLC27A3 | Solute carrier family 27 (fatty acid transporter), member 3 | -4.08 |
| SLC27A4 | Solute carrier family 27 (fatty acid transporter), member 4 | -2.04 |
| SLC27A5 | Solute carrier family 27 (fatty acid transporter), member 5 | 12.92 |
| SLC27A6 | Solute carrier family 27 (fatty acid transporter), member 6 | -29.92 |
